# miR-107 reverses the multidrug resistance of gastric cancer by targeting the CGA/EGFR/GATA2 positive feedback circuit

**DOI:** 10.1016/j.jbc.2024.107522

**Published:** 2024-07-02

**Authors:** Pei Wang, Yelu Zhou, Juan Wang, Yun Zhou, Xiaohui Zhang, Yanxing Liu, Ang Li, Yangsong He, Shuyi Chen, Airong Qian, Xin Wang, Yongzhan Nie, Daiming Fan, Tianyu Cao, Yuanyuan Lu, Xiaodi Zhao

**Affiliations:** 1 Xijing Hospital of Digestive Diseases, State Key Laboratory of Holistic Integrative Management of Gastrointestinal Cancers and National Clinical Research Center for Digestive Diseases, Fourth Military Medical University, Xi’an, Shaanxi, China; 2 Department of Gastroenterology, Tangdu Hospital, Fourth Military Medical University, Xi’an, Shaanxi, China; 3 School of Basic Medical Sciences, Fourth Military Medical University, Xi’an, Shaanxi, China; 4 Key Laboratory for Space Biosciences and Biotechnology, School of Life Sciences, Northwestern Polytechnical University, Xi’an, Shaanxi, China

**Keywords:** gastric cancer, multidrug resistance, miRNA, CGA, GATA2, EGFR signaling

## Abstract

Chemotherapy is still the main therapeutic strategy for gastric cancer (GC). However, most patients eventually acquire multidrug resistance (MDR). Hyperactivation of the EGFR signaling pathway contributes to MDR by promoting cancer cell proliferation and inhibiting apoptosis. We previously identified the secreted protein CGA as a novel ligand of EGFR and revealed a CGA/EGFR/GATA2 positive feedback circuit that confers MDR in GC. Herein, we outline a microRNA-based treatment approach for MDR reversal that targets both CGA and GATA2. We observed increased expression of CGA and GATA2 and increased activation of EGFR in GC samples. Bioinformatic analysis revealed that miR-107 could simultaneously target CGA and GATA2, and the low expression of miR-107 was correlated with poor prognosis in GC patients. The direct interactions between miR-107 and CGA or GATA2 were validated by luciferase reporter assays and Western blot analysis. Overexpression of miR-107 in MDR GC cells increased their susceptibility to chemotherapeutic agents, including fluorouracil, adriamycin, and vincristine, *in vitro*. Notably, intratumor injection of the miR-107 prodrug enhanced MDR xenograft sensitivity to chemotherapies *in vivo*. Molecularly, targeting CGA and GATA2 with miR-107 inhibited EGFR downstream signaling, as evidenced by the reduced phosphorylation of ERK and AKT. These results suggest that miR-107 may contribute to the development of a promising therapeutic approach for the treatment of MDR in GC.

Gastric cancer (GC) is a highly lethal type of gastrointestinal cancer that is associated with exceptionally high rates of morbidity and mortality and poses tremendous health challenges worldwide (1). Despite significant advancements in cancer treatment methods in recent decades, chemotherapy remains the primary approach for the treatment of GC (2). The use of chemotherapy regimens such as FOLFOX and CAPOX, which contain fluorouracil and oxaliplatin, has significantly increased the 5-years survival rate of GC patients by 10%, making them the preferred first-line treatment options for advanced disease (3). However, the occurrence of multidrug resistance (MDR) is observed during cancer chemotherapy. MDR is the ability of cancer cells to evade the effects of a multitude of anticancer drugs which has become one of the main impediments to the success of cancer chemotherapy (4, 5). Thus, identification of the mechanisms by which GC cells acquire MDR is important for developing novel therapeutic strategies and improving the therapeutic efficacy of GC patients.

The epidermal growth factor receptor (EGFR) signaling pathway plays a crucial role in regulating cellular growth, survival, proliferation, and differentiation, making it an indispensable component of the normal functions of cells (6). The activation of EGFR is initiated by specific ligands, leading to the activation of a multitude of intracellular signaling pathways, notably the ERK and AKT pathways (7). Abnormal activation of the EGFR pathway has been reported to be associated with chemoresistance in many types of cancer (8, 9, 10, 11). We recently identified the glycoprotein hormone α subunit (CGA) as a novel ligand for EGFR (12). Increased CGA expression in GC cells activates the EGFR signaling pathway, leading to the development of MDR. The transcription factor GATA2 plays a crucial role in regulating CGA expression, and the activation of EGFR caused by CGA in turn increases GATA2 expression, thus resulting in a positive feedback loop between CGA, EGFR, and GATA2. Although tyrosine kinase inhibitors and monoclonal antibodies targeting EGFR have been widely used (13), interfering with the EGFR/GATA2/CGA circuit by targeting either GATA2 or CGA could effectively prevent EGFR activation and potentially overcome drug resistance, which warrants further exploration.

miRNAs are a class of single-stranded, noncoding RNAs that can exert their effects by inhibiting protein translation or promoting RNA degradation through interactions with the 3′-UTRs of their target mRNAs (14). With their inherent roles in gene regulation, these potential drug targets hold immense promise and could be a promising avenue for future drug development (15). Since miRNA‒mRNA interactions do not require perfect pairing, one miRNA strand can recognize multiple targets (16). Moreover, miRNA therapies present a unique advantage in targeting "nondruggable" proteins, which are conventionally inaccessible to traditional small molecule drugs due to a lack of enzymatic function or limited conformational accessibility (17). Recent research has suggested that miRNAs are involved in the development of MDR, a phenomenon that has a positive influence on treatment efficacy. For instance, miR-145 restores the sensitivity of colorectal cancer to oxaliplatin by inhibiting the expression of its target GPR98 (18). Our previous investigation demonstrated that miR-135b-5p plays a crucial role in decreasing resistance to chemotherapy and triggering apoptosis in GC cells by specifically targeting the integrin 2 protein ITGA2 (19). Therefore, miRNAs offer a promising avenue for the development of novel therapeutics for the treatment of MDR.

Our study presents a novel miRNA-based therapeutic approach to reverse drug resistance in GC by targeting the CGA/EGFR/GATA2 circuit. We identified miR-107 as a potent regulator of both CGA and GATA2, effectively suppressing their activity. Administration of miR-107 has been shown to enhance the sensitivity of both MDR GC cells and xenografts to chemotherapeutic agents both *in vitro* and *in vivo*. Mechanistically, targeting the *CGA* and *GATA2* genes with miR-107 inhibited downstream EGFR signaling pathways, ultimately leading to the suppression of associated cellular processes. Overall, our study provides compelling evidence suggesting that miR-107 could function as a potent inhibitor of the CGA/EGFR/GATA2 circuit and is therefore a promising candidate for the development of miRNA drugs that could improve the effectiveness of chemotherapy.

## Results

### Screening for miRNAs that target the CGA/EGFR/GATA2 circuit in GC cells

**Figure 1:**
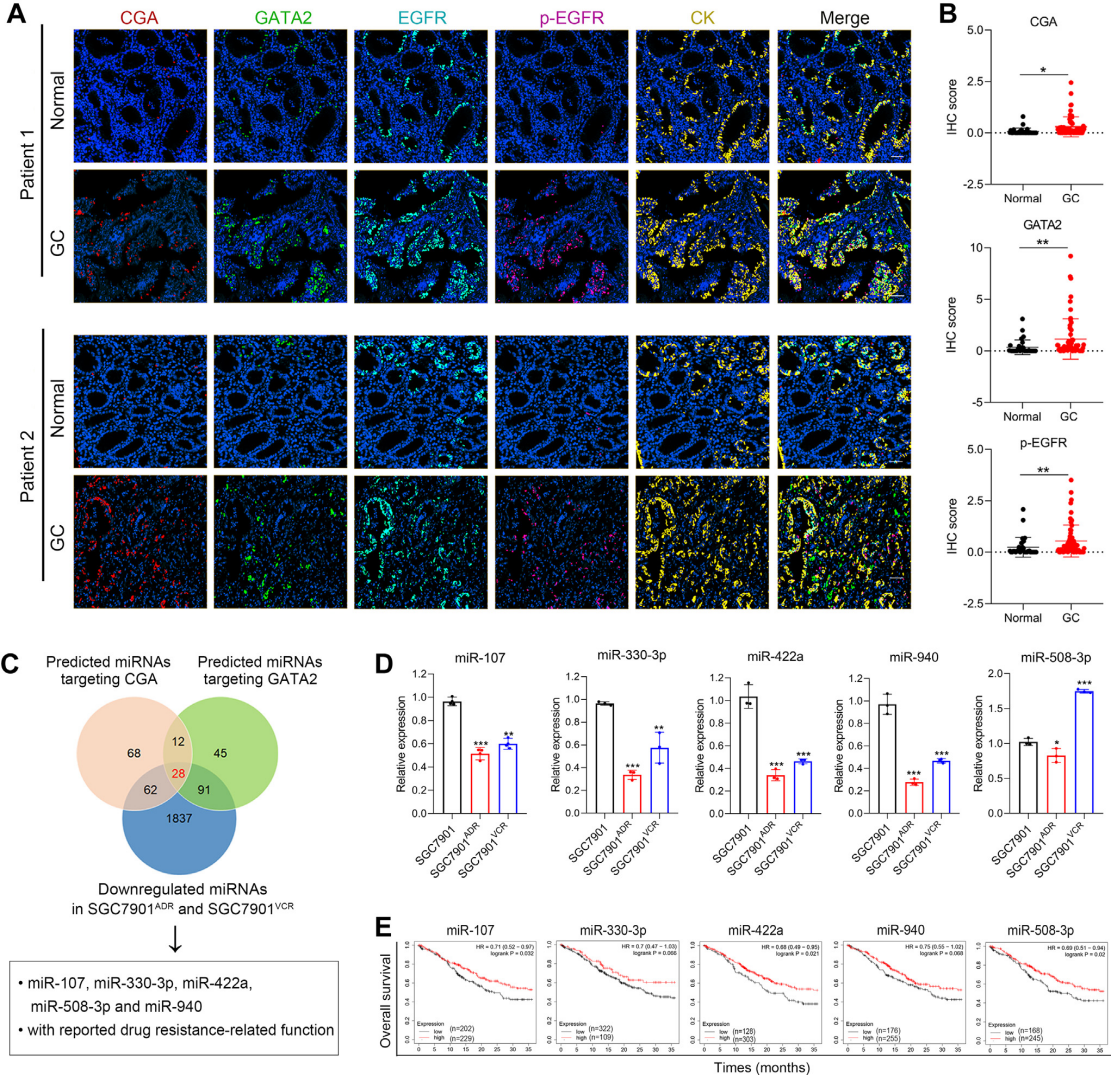
**Validation of the CGA/EGFR/GATA2 positive feedback loop in GC patients and screening for miRNAs targeting CGA and GATA2.***A*, multiplex immunohistochemistry of CGA, GATA2, EGFR, p-EGFR, and CK in two paired GC patients. *B*, statistical analysis of CGA, GATA2, and p-EGFR expression in GC tissues and adjacent normal tissues (n = 20). *C*, diagram of screening for CGA- and GATA2-targeting miRNAs. *D*, expression levels of candidate miRNAs measured by RT‒PCR (n = 3), the data in the bar plots are expressed as the mean ± S.D. *E*, Kaplan-Meier (https://kmplot.com/analysis/) analyses of correlations between the expression of candidate miRNAs and overall survival of GC patients. Scale bars represent 20 μm. Significant differences were assessed among multiple groups using one-way ANOVA (*D*) and between two groups using a *t* test (*B*). ∗*p*< 0.05, ∗∗*p*< 0.01. CI, confidence interval; GC, gastric cancer; HR, hazard ratio.

To examine the activity of the CGA/EGFR/GATA2 circuit in patients who received chemotherapy, multiplex immunofluorescence analysis was performed on a tissue microarray comprising 20 paired GC tissues and adjacent normal tissues from patients undergoing adjuvant chemotherapy. The results revealed significantly greater levels of CGA, GATA2, and p-EGFR in the GC tissues than in the corresponding normal tissues (Fig. 1, *A* and *B*). These findings support the activation of the CGA/EGFR/GATA2 circuit in GC patients undergoing chemotherapy. Previously, we demonstrated that GATA2 is upregulated by chemotherapy and transactivates CGA expression and secretion in GC cells, leading to hyperactivation of the EGFR pathway (12). Given the importance of miRNAs in regulating multiple targets at the posttranscriptional level, we sought to identify miRNAs that target both CGA and GATA2 simultaneously. Using multiple bioinformatic algorithms, we identified 170 miRNAs targeting CGA and 176 miRNAs targeting GATA2. Among these candidates, 28 miRNAs targeted both CGA and GATA2 and were downregulated in SGC7901^ADR^ and SGC7901^VCR^ cells compared to the parental SGC7901 cells (20) (Fig. 1*C*). Furthermore, five of the 28 miRNAs, including miR-107, miR-330-3p, miR-422a, miR-508-3p, and miR-940, were selected for further analyses because they are known to be involved in chemoresistance or tumor suppression (21, 22, 23, 24, 25). RT‒PCR analysis was conducted to validate the expression of the five miRNAs, and the results revealed that four miRNAs (miR-107, miR-330-3p, miR-422a, and miR-940) were downregulated in SGC7901^ADR^ and SGC7901^VCR^ cells compared with SGC7901 cells (Fig. 1*D*). Then Kaplan‒Meier database was used to analyze the correlations between candidate miRNAs and overall survival of GC patients. Results identified that the low expression of miR-107, miR-422a, and miR-508-3p was associated with poor prognosis in GC patients (*p* < 0.05) (Fig. 1*E*). Together, these results suggest that the miR-107 and miR-422a potentially target CGA and GATA2 in GC cells.

### miR-107 directly targets CGA and GATA2 in GC cells

**Figure 2:**
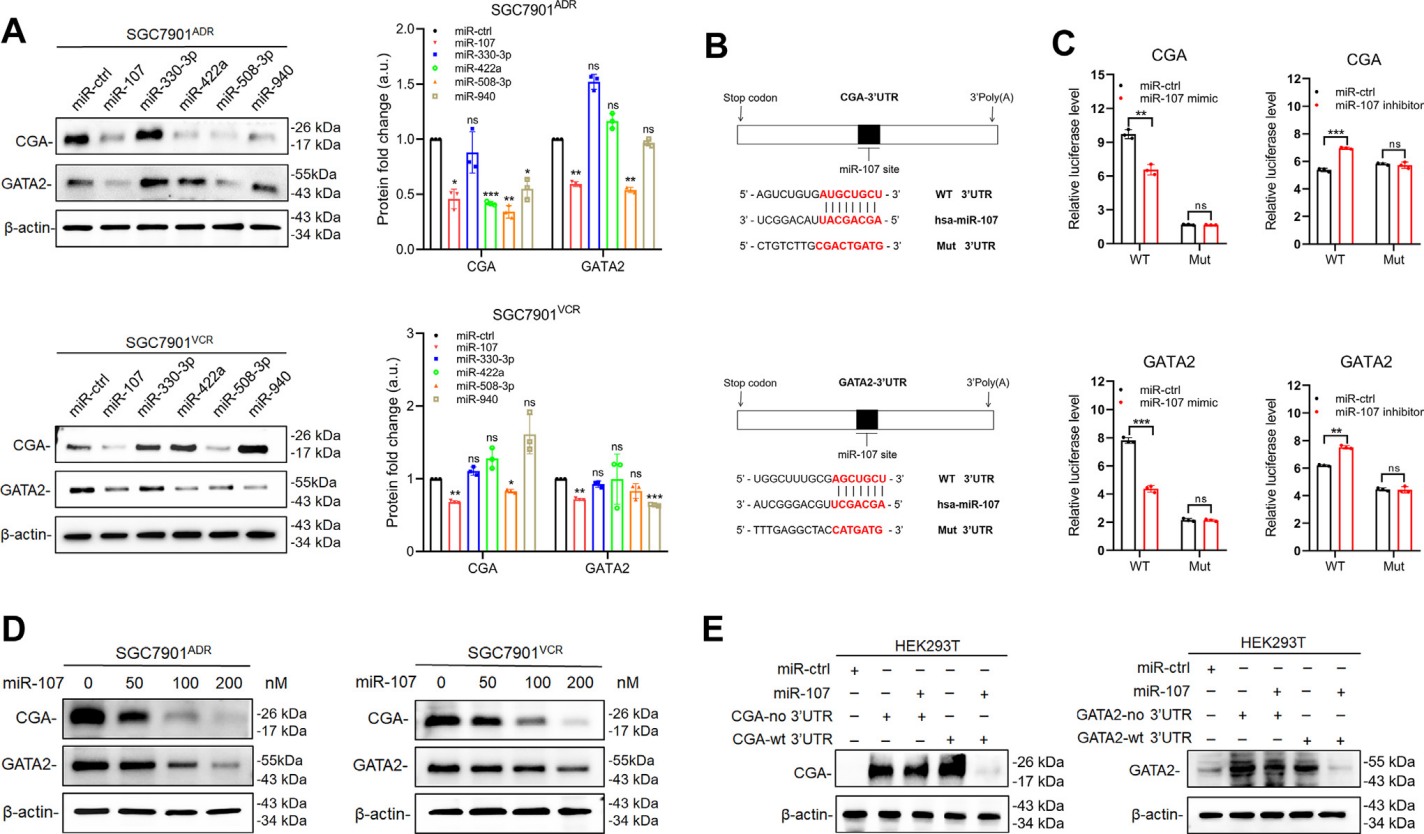
**miR-107 directly targets CGA and GATA2 in MDR GC cells.***A*, immunoblots and quantitative analysis of the CGA and GATA2 proteins in SGC7901^ADR^ and SGC7901^VCR^ cells. Blots are representative of three independent experiments. a.u., arbitrary unit. The data are presented as the means ± S.D.s from three independent experiments. *B*, predicted binding sites of miR-107 in the 3′UTR of CGA and GATA2 mRNA. *C*, relative luciferase reporter activity in HEK293T cells cotransfected with WT or mutated (Mut) reporter plasmids and miR-107, miR-107 inhibitor, and their controls. *D*, protein expression of CGA and GATA2 after treatment with different concentrations of miR-107 in SGC7901^ADR^ and SGC7901^VCR^ cells. *E*, protein expression of CGA and GATA2 after transfection of the CGA or GATA2 plasmid containing the WT 3′UTR or lacking the 3′UTR (no 3′UTR) along with miR-107 or miR-ctrl in HEK293T cells. The data in the bar plots are expressed as the mean ± S.D. Significant differences were assessed by using one-way ANOVA (*A*) and the *t* test (*C*). ∗*p*< 0.05, ∗∗*p*< 0.01, ∗∗∗*p*< 0.001. ns, not significant.

To identify the suppressive impact of these miRNA candidates on the protein expression of CGA and GATA2, we transfected SGC7901^ADR^ and SGC7901^VCR^ cells with miRNA mimics of miR-107, miR-330-3p, miR-422a, miR-508-3p, and miR-940. Western blotting results revealed that the protein levels of CGA and GATA2 exhibited the greatest reduction after transfection with miR-107 in both MDR cell lines (Fig. 2*A*), indicating that miR-107 exhibits promising therapeutic potential for interrupting the CGA/EGFR/GATA2 circuit. In addition, the sequencing data we previously obtained revealed that miR-107 was expressed at lower levels in drug-resistant cells than in GC cells (20), and two sample sets (GSE30070 and GSE49052) from the GEO (https://www.ncbi.nlm.nih.gov/geo/) also supported our results; detailed information is provided in Fig. S1. To validate the interaction between miR-107 and CGA or GATA2, we analyzed the 3′-UTR sequences of CGA and GATA2 mRNA and discovered a possible binding site for miR-107 (Fig. 2*B*). Subsequently, to determine whether CGA or GATA2 was a direct target of miR-107, fragments of the CGA and GATA2 3′-UTR sequences, which contained either wild type (WT) or mutant-binding sites, and miR-107 or miR-107 inhibitors were inserted into the region of the luciferase reporter gene. Luciferase reporter assays revealed that miR-107 caused a considerable decrease in the relative luciferase activity of HEK293T cells with the WT 3′-UTR of GATA2 or CGA but not in cells with the mutant 3′-UTR. In contrast, the miR-107 inhibitor increased the luciferase activity of HEK293T cells harboring the WT 3′-UTR of CGA or GATA2 (Fig. 2*C*). Moreover, the protein levels of CGA and GATA2 decreased in a dose-dependent manner upon miR-107 transfection into SGC7901^ADR^ and SGC7901^VCR^ cells (Fig. 2*D*). Next, we transfected HEK293T cells with a CGA or GATA2 plasmid with or without their 3′-UTRs. When miR-107 was transfected into these cells, CGA and GATA2 expression were markedly reduced in the cells that were transfected with the plasmids containing the 3′-UTRs but not in the cells lacking the 3′-UTRs (Fig. 2*E*). Together, these results indicated that miR-107 can inhibit the expression of GATA2 and CGA by directly targeting their 3′-UTRs.

### miR-107 sensitizes MDR GC cells to chemotherapeutic drugs *in vitro*

**Figure 3:**
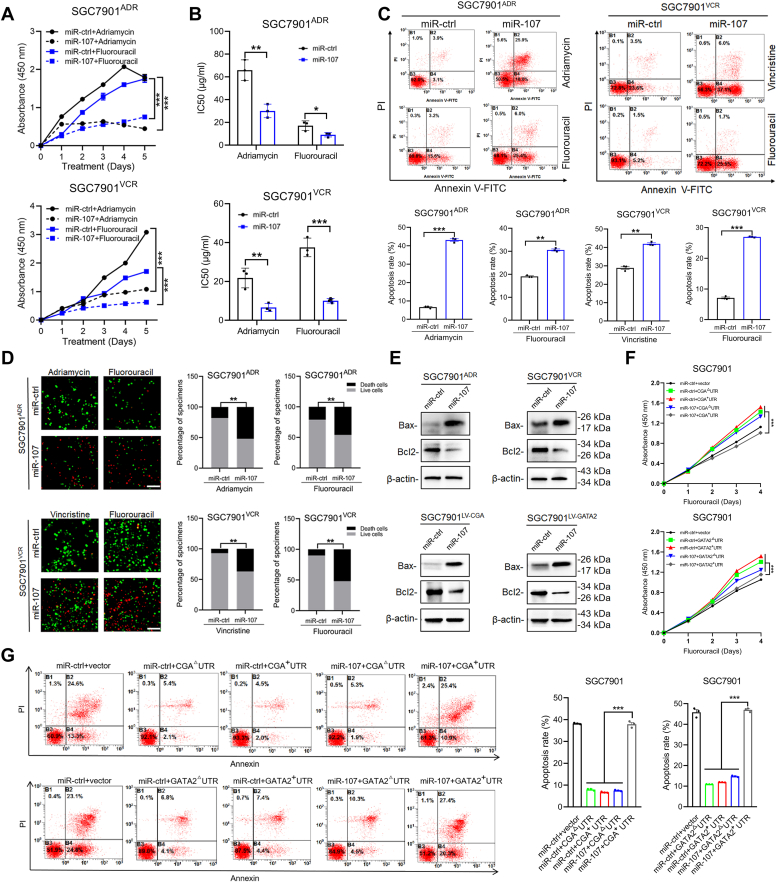
**miR-107 sensitizes MDR GC cells to chemotherapeutic drugs *in vitro.****A*, proliferation of SGC7901^ADR^ and SGC7901^VCR^ cells after transfection with miR-107 or miR-ctrl in the presence of chemotherapeutic drugs. *B*, IC50 values in SGC7901^ADR^ and SGC7901^VCR^ cells after transfection with miR-107 or miR-ctrl in the presence of chemotherapeutic drugs. *C* and *D*, apoptosis of SGC7901^ADR^ and SGC7901^VCR^ cells after transfection with miR-107 or miR-ctrl in the presence of chemotherapeutic drugs. *E*, Western blot analysis of Bax and Bcl2 in SGC7901^ADR^ and SGC7901^VCR^ cells (*upper*) or SGC7901 cells stably expressing CGA or GATA2 (*lower*) after transfection with miR-107 or miR-ctrl. *F*, proliferation of SGC7901 cells after transfection with miR-107, miR-ctrl, CGA, or the GATA2 vector in the presence of chemotherapeutic drugs. *G*, apoptosis of SGC7901 cells after transfection with miR-107, miR-ctrl, and CGA and GATA2 vector in the presence of chemotherapeutic drugs. The data in the bar plots are expressed as the mean ± S.D. (n = 3). Significant differences were assessed in multiple groups using one-way ANOVA (*G*) or repeated-measures ANOVA (*A* and *F*) and in two groups using the *t* test (*B* and *C*). ∗*p*< 0.05, ∗∗*p*< 0.01, ∗∗∗*p*< 0.001. Scale bar represents 50 μm.

To investigate the influence of miR-107 on chemoresistance, we examined the impact of miR-107 on regulating the level of resistance to various chemotherapeutic drugs used for GC treatment, including fluorouracil, adriamycin, and vincristine. The SGC7901^ADR^ and SGC7901^VCR^ cell lines exhibited varying degrees of cross-resistance to these drugs, providing insights into the potential effect of miR-107 on the regulation of chemoresistance. First, we investigated the proliferation and apoptosis ability of cells by transfecting miR-107 into cells. It was concluded that the proliferation of cells was significantly inhibited by treatment with miR-107 and chemotherapeutic agents (Fig. 3*A*). Then, IC50 assay was employed to evaluate the resistance of MDR gastric cells to various chemotherapeutic agents. The results indicated that miR-107 could reduce the IC50 values of chemotherapeutic agents in SGC7901^ADR^ and SGC7901^VCR^ cells (Fig. 3*B*), which demonstrated that miR-107 could restore the resistance of MDR GC cells to various chemotherapeutic agents. Subsequently, flow cytometry analysis revealed that miR-107 successfully promoted apoptosis in SGC7901^ADR^ and SGC7901^VCR^ cells upon exposure to chemotherapeutic drugs (Fig. 3*C*). Consistent findings from the LIVE/DEAD viability analysis demonstrated that miR-107 significantly enhanced the efficacy of chemotherapeutic drugs in inducing cell apoptosis (Fig. 3*D*). We further elucidated the impact of miR-107 on molecules that regulate apoptosis and discovered that its overexpression led to a significant increase in the expression of the proapoptotic protein Bax, while it had a profound effect on reducing the expression of the antiapoptotic protein Bcl-2 in SGC7901^ADR^ and SGC7901^VCR^ cells (Fig. 3*E*). In addition, cell proliferative ability and cell apoptosis were also determined without drug treatment. It was found that miR-107 affected the proliferation and apoptosis of SGC7901^ADR^ and SGC7901^VCR^ in a dose-dependent manner. Notably, the inhibitory effect on proliferation and apoptosis by SGC7901 were not significantly observed when compared with two chemoresistant cell lines. (Fig. S2). The results confirmed that the function of miR-107 affecting cell proliferation and apoptosis depended on high CGA and GATA2 expression. In addition, to verify the relationship between miR-107 and this axis, rescue experiments were performed in SGC7901 cells. We observed that overexpression of the CGA 3′UTR or GATA2 3′UTR promoted chemoresistance and decreased apoptosis, and these effects were reversed by miR-107 supplementation (Fig. 3, *F* and *G*). These results suggested that miR-107 was responsible for overcoming MDR *via* its targets CGA and GATA2 in GC cells.

### miR-107 sensitizes MDR GC xenografts to chemotherapeutic drugs *in vivo*

**Figure 4:**
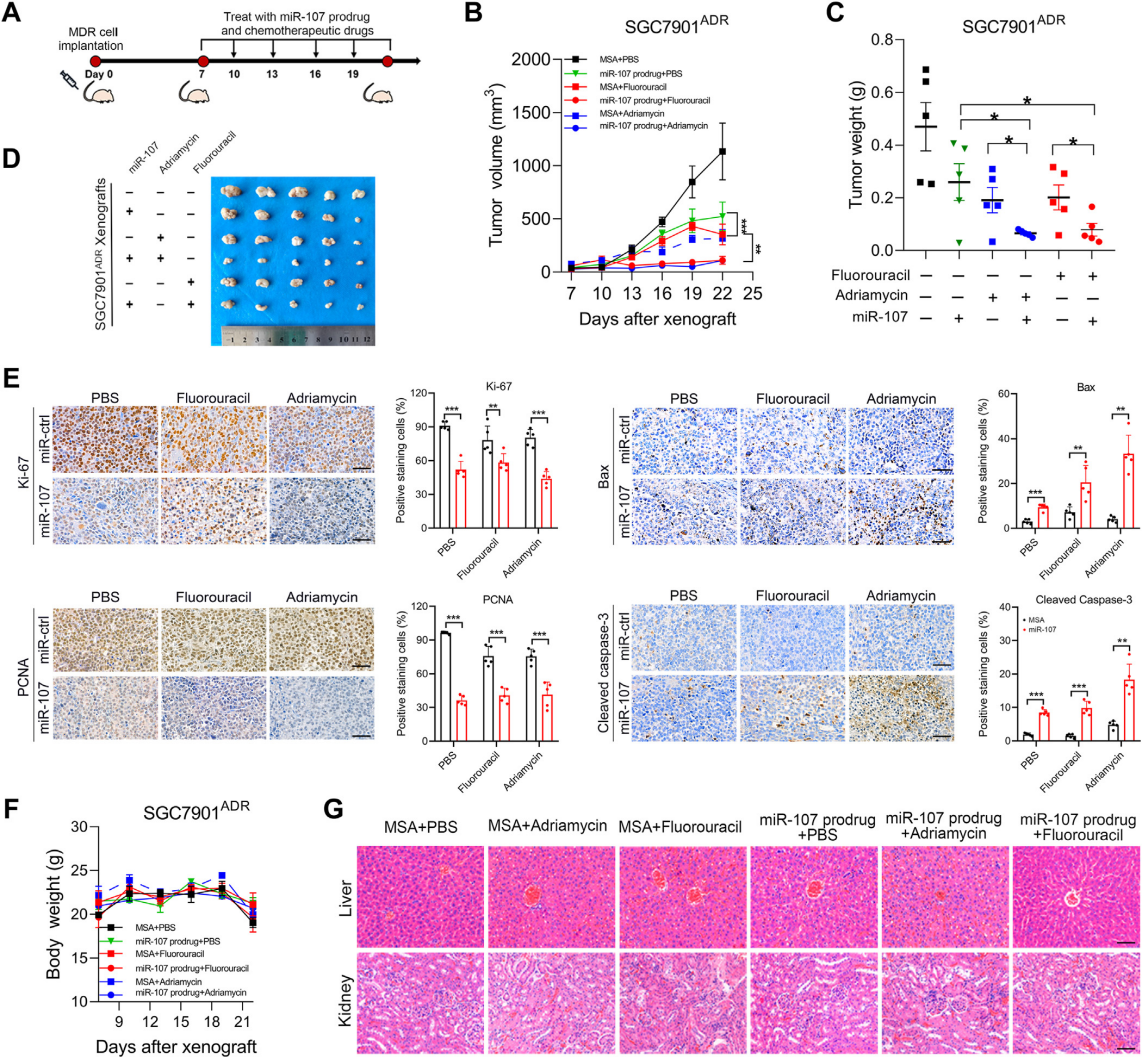
**miR-107 sensitizes MDR xenografts to chemotherapeutic drugs *in vivo*.***A*-*C*, nude mice (n = 5) were subcutaneously implanted with SGC7901^ADR^ cells. When the tumor size reached 100 mm^3^, the mice received the indicated treatment every 3 days: (*A*) adriamycin, 8 mg/kg, i.p. injection; fluorouracil, 20 mg/kg, i.p. injection; miR-107 prodrug, 1 nmol/mouse intratumoral injection. Tumor volume (*B*) and tumor weight (*C*) were measured. *D*, representative images of tumors from each group. *E*, representative IHC staining of Ki67, PCNA, Bax, and cleaved Caspase-3 in xenografts harvested from the indicated groups. Scale bar represents 50 μm. The percentages of staining-positive cells were measured. *F*, mouse body weights for each group were recorded. *G*, representative H&E staining images of xenografts, livers, and kidneys from each group. Scale bars represent 50 μm. The data in the bar plots are expressed as the mean ± S.D, (*E*) or mean ± S.E.M. (*B*, *C*, and *F*) (n = 5). Significant differences were assessed in multiple groups using repeated-measures ANOVA test (*B*) or one-way ANOVA test (*C*) and two groups using the *t* test (*E*). ∗*p*< 0.05, ∗∗*p*< 0.01, ∗∗∗*p*< 0.001. MSA, negative control of the miR-107 prodrug. IHC, immunohistochemistry.

To assess the efficacy of miR-107 in overcoming chemoresistance, we developed a chimeric miR-107 agent (namely the miR-107 prodrug) and a specific control for the miR-107 prodrug, the Sephadex aptamer-tagged methionyl-tRNA scaffold (tRNA/MSA), using a Sephadex aptamer-tagged methionyl-tRNA scaffold-based approach, which enables miRNAs to capture cellular mRNA with natural characteristics (26, 27, 28). SGC7901^ADR^ cells were subcutaneously implanted into nude mice, followed by treatment with both chemotherapy drugs and the miR-107 prodrug (Fig. 4*A*). Although chemotherapy or intratumoral injection of the miR-107 prodrug reduced tumor growth to some extent, the combination of the miR-107 prodrug with fluorouracil or adriamycin elicited more notable tumor shrinkage (Fig. 4, *B*–*D*). Moreover, immunohistochemistry demonstrated that the miR-107 prodrug inhibited cell proliferation and caused apoptosis in xenografts exposed to chemotherapy (Fig. 4*E*). The mice treated with the miR-107 prodrug exhibited no weight loss or signs of liver or kidney abnormalities (Fig. 4, *F* and *G*). These findings indicate that miR-107 renders MDR GC xenografts more susceptible to chemotherapy and provides a promising therapeutic approach for treating MDR.

### miR-107 inhibits signaling pathways downstream of EGFR

**Figure 5:**
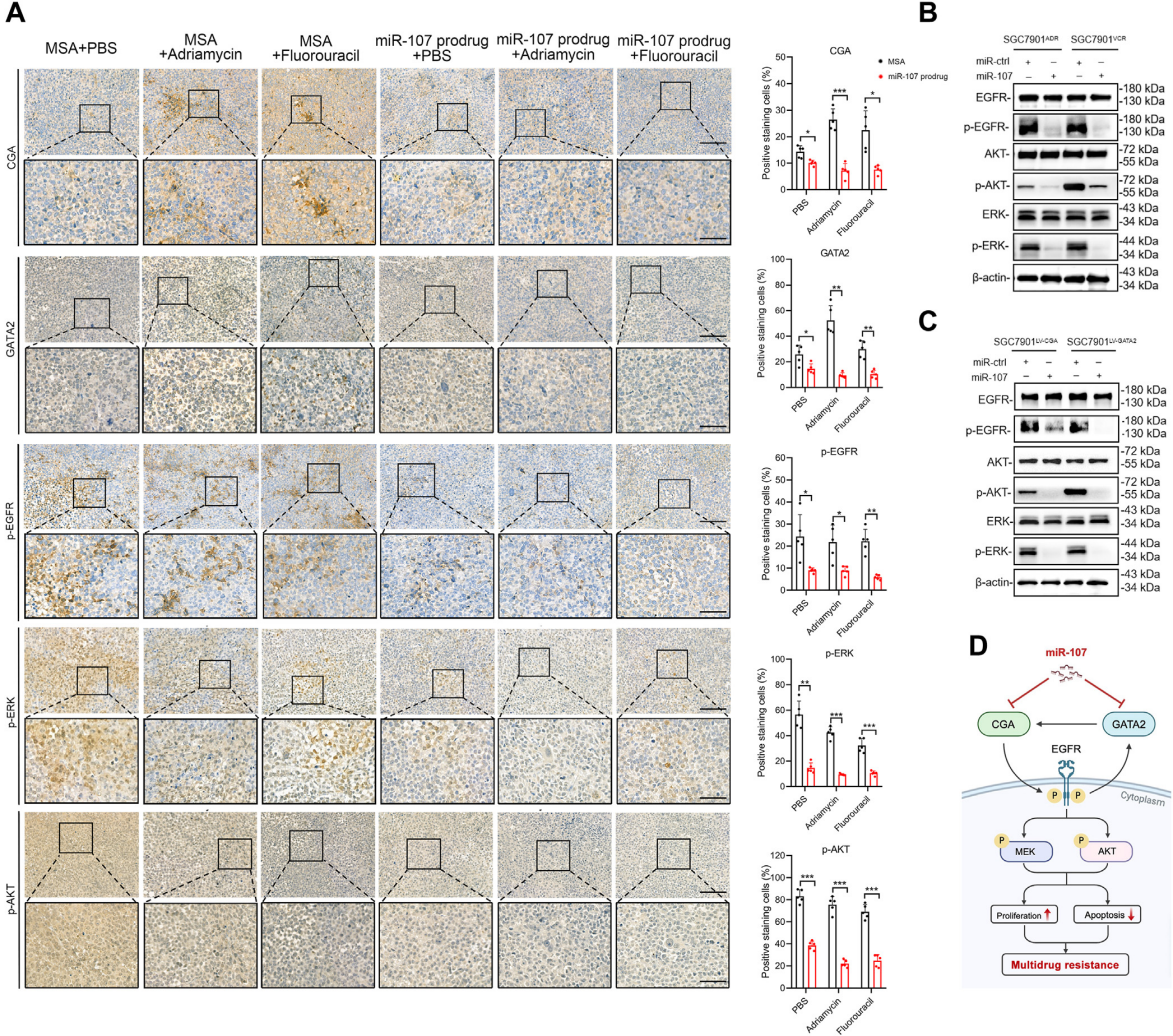
**miR-107 inhibits EGFR downstream signaling in MDR GC cells and xenografts.***A*, IHC staining of CGA, GATA2, p-EGFR, p-ERK, and p-AKT in xenografts from mice given the indicated treatments. The percentages of staining-positive cells were measured. *B* and *C*, Western blotting analysis of total and phosphorylated EGFR, AKT, and ERK after transfection of GC cells with miR-107 or miR-ctrl. *D*, proposed mechanism model in this study, drawn from the website “https://www.biorender.com/”. Scale bars represent the following: upper, 20 μm; lower, 50 μm. The data in the bar plots are expressed as the mean ± S.D. (n = 5). Significant differences were assessed by the *t* test (*A*). ∗*p*< 0.05, ∗∗*p*< 0.01, ∗∗∗*p*< 0.001. IHC, immunohistochemistry; ns, not significant.

To determine the role of miR-107 in reversing MDR in GC cells, we investigated the GATA2/CGA axis-mediated activation of the EGFR signaling pathway. The results demonstrated that miR-107–based treatments alone or in combination with chemotherapy not only diminished GATA2 and CGA expression but also effectively inhibited phosphorylated EGFR and its downstream phosphorylated ERK and AKT (Fig. 5*A*). Correspondingly, miR-107 suppressed the phosphorylation of EGFR, ERK, and AKT in SGC7901^ADR^ and SGC7901^VCR^ cells (Fig. 5*B*). Similar findings were observed in SGC7901 cells overexpressing GATA2 or CGA together with miR-107 (Fig. 5*C*). The proposed working model in our study is shown in Figure 5*D*. Taken together, these findings suggest that miR-107 reverses MDR in GC cells by directly targeting CGA and GATA2, ultimately resulting in the repression of EGFR and its downstream signaling pathways.

## Discussion

The poor prognosis of GC patients is largely attributed to MDR, a significant contributor to cancer progression. Our primary objective in this study was to gain deeper insights into the regulatory mechanism of the previously discovered CGA/EGFR/GATA2 feedback loop, which is a critical factor in the pathogenesis of MDR. Our analysis revealed that miR-107 is a potential therapeutic target that directly modulates both CGA and GATA2, two essential components of this positive feedback loop. Importantly, miR-107 treatment was found to restore the sensitivity of MDR gastric carcinoma cells to multiple chemotherapeutic agents both *in vitro* and *in vivo*, which suggests that miR-107 may be an effective miRNA drug candidate. Mechanistically, our investigation demonstrated that miR-107 acts as an inhibitor of EGFR and its downstream signaling pathways. Our study revealed miR-107 as a novel miRNA drug candidate for overcoming MDR in GC.

Our previous study demonstrated that CGA and GATA2 play crucial roles in promoting MDR in GC. Upon sublethal exposure to chemotherapy drugs, GATA2 is phosphorylated and then initiates its autoregulation, leading to the secretion of CGA. CGA accumulation can trigger hyperactivation of the EGFR signaling pathway (12). Our current study revealed that CGA and GATA2 levels are elevated in GC patients. Therefore, CGA and GATA2 serve as valuable therapeutic targets to prevent and reverse chemoresistance. Given the reciprocal regulation between CGA and GATA2, inhibition of either may result in compensatory effects on the other. Consequently, blocking both CGA and GATA2 simultaneously is a feasible approach. Our study revealed that miR-107 directly targets CGA and GATA2, promoting chemotherapy-induced apoptosis and restoring chemosensitivity to MDR GC cells. These findings suggest that targeting CGA and GATA2 through miR-107 inhibition is a promising strategy for overcoming MDR in GC.

The strategy that we used to screen miRNAs that target CGA and GATA2 is highly reliable and convincing. First, we identified 40 miRNA candidates by overlapping the predicted miRNAs targeting CGA or GATA2 from various algorithms, including DIANAmT, miRanda, miRDB, miRWalk, RNAhybrid, PICTAR, PITA, and RNA22. This approach greatly reduced the false-positive rate of these prediction programs, resulting in increased accuracy of miRNA identification. Compared with our previously published RNA-seq data (20), 28 miRNAs were downregulated in MDR cells. This suggests that these miRNAs may be potential suppressors of chemoresistance. Furthermore, five miRNAs have been identified as either playing a role in chemoresistance or being associated with patient prognosis, adding to their potential significance as targets for therapeutic intervention. Finally, miR-107 was identified due to its significant impact on repressing CGA and GATA2 at the protein level, and it was confirmed to bind to both of their 3′-UTRs. To investigate the possibility of cancer-promoting gene mutations in GC MDR, PCR amplification and Sanger sequencing were performed on all exons of CGA, GATA2, and EGFR in the cell lines we used. We concluded that none of the mutations had any effect on MDR, as shown in Table S1. Previously, miR-107 was shown to increase sensitivity to chemotherapy drugs in various cancers, including breast cancer, colorectal cancer, liver cancer, and glioma (21, 29, 30, 31). Our study provides valuable evidence that the delivery of miR-107 could serve as a potential therapeutic approach to overcome MDR in GC.

Multiple approaches have been developed to generate therapeutic miRNAs for cancer treatment *in vivo*. Chemical synthesis and modifications have been widely applied to formulate miRNA drugs for biological investigation (32). Despite efforts to overcome these challenges, two major obstacles remain in the development of miRNA drugs, namely, off-target effects and immunogenicity, leading to reduced efficiency and potentially hazardous side effects (33, 34). Our research team and collaborators have developed a hybrid miR-107 and tRNA Met-fused Sephadex aptamer, which provides a solution by preventing off-target effects and immunogenicity while greatly enhancing natural miRNA production (26, 27, 28). *In vivo* experiments demonstrated that the miR-107 prodrug provided a satisfactory inhibitory effect on tumor growth and that miR-107 combined with chemotherapy caused minimal side effects, indicating the promising potential of miR-107 for restoring chemosensitivity in GC.

Our study demonstrated that miR-107 can reverse drug resistance in GC cells by directly targeting and suppressing CGA and GATA2 expression. This, in turn, leads to the downregulation of EGFR and its downstream signaling pathways. This novel miRNA-based therapeutic strategy holds great promise for enhancing the efficacy of chemotherapy in the treatment of GC patients.

## Experimental procedures

### Human tissue samples

The GC tissue microarrays comprised 20 paired samples of primary GC and adjacent nontumor epithelial tissues collected from Xijing Hospital of Digestive Diseases. All patients provided informed consent and this study was approved by Xijing Hospital’s Protection of Human Subjects Committee. The study abided by the Declaration of Helsinki principles. The detailed information of the 20 patients is provided in Table S2.

### Cell culture

The human GC cell line SGC7901 was obtained from the State Key Laboratory of Cancer Biology (CBSKL). MDR SGC7901^VCR^ and SGC7901^ADR^ cells, which were derived from SGC7901 cells, were generated as previously described (35, 36, 37). Briefly, SGC7901 cells were harvested during the logarithmic growth phase and subsequently cultivated in complete culture medium supplemented with either vincristine or adriamycin at a concentration of 0.2 μg/ml. After a two- to three-day interval, the fresh drug-containing culture medium was replaced. Over time, the concentration of vincristine gradually increased until it reached 0.8 to 1.0 μg/ml, and the adriamycin concentration similarly increased until it reached 0.5 μg/ml. After 3 months of continuous culture, the drug-resistant cell lines SGC7901^VCR^ and SGC7901^ADR^ were obtained. To maintain the long-term MDR phenotype, adriamycin and vincristine at final concentrations of 1 μg/ml and 0.5 μg/ml were added to the SGC7901^ADR^ and SGC7901^VCR^ cell culture media, respectively. The cells were cultivated in an enriched environment consisting of Dulbecco's modified Eagle's medium (Gibco) supplemented with 10% fetal bovine serum (Gibco), 100 U/ml penicillin, and 100 mg/ml streptomycin. This environment was also controlled at 5% carbon dioxide and a temperature of 37 °C to maintain optimal conditions for cell growth and development.

### Plasmid construction and cell transfection

Plasmid construction and cell transfection were conducted as previously described (12). To recap, the CGA-WT and CGA-3′-UTR plasmids, along with the GATA2-WT and GATA2-3′-UTR plasmids, were cloned downstream of the *CGA* and *GATA2* genes in the pcDNA3.1 vector utilizing the EcoR and Hind III sites for insertion. DNA sequencing was used to verify the successful construction of these plasmids. miR-107, miR-330-3p, miR-422a, miR-508-3p, and miR-940 mimics, as well as miR-ctrl, were synthesized by RiboBio. GP transfect-mate was used to deliver these mimics and miR-ctrl into cells. Transient transfection of the plasmids (2 μg) into the cells was performed with the aforementioned reagents. After 48 h, the transfected cells were used for further experimentation or analysis.

### Real-time PCR

Total microRNA was extracted from cells using the miRNeasy Mini Kit (Qiagen) in accordance with the manufacturer’s instructions, and microRNA was reverse transcribed into cDNA by using the PrimeScript RT reagent kit (TaKaRa). Then, qPCR was performed in triplicate using SYBR Premix Ex TaqTM II (TaKaRa). The primers used for mature miR-107, miR-330-3p, miR-422a, miR-508-3p, miR-940, and U6 were designed and synthesized by TSINGKE. PCR amplification was conducted with the following cycling conditions: 95 °C for 30 s, 40 cycles of 95 °C for 5 s, and 60 °C for 30 s, with a final extension at 95 °C for 10 s, 65 °C for 5 s, and 95 °C for 5 s on a CFX96 Real-Time PCR Detection System (Bio-Rad). The 2^−ΔΔCT^ method was used to determine RNA expression levels between groups, with U6 small nuclear RNA serving as an internal control. The detailed primer information is provided in Table S3.

### Western blotting

Cultured cells were lysed with RIPA buffer containing protease and phosphatase inhibitors, and the proteins were subsequently extracted. To denature the proteins, 5 × loading buffer was used at 100 °C for 10 min. After electrophoresis on 10% SDS‒PAGE gels, the proteins were transferred onto nitrocellulose membranes, which were then blocked with 1× fast blocking solution at room temperature for 15 min. Primary and secondary antibodies were incubated with the membranes, which were scanned and quantitatively analyzed using a Molecular Imager ChemiDox XRS + Imaging System with Image Lab software (Bio-Rad, https://www.bio-rad.com/zh-cn/sku/1709690-image-lab-software?ID=1709690). Primary antibodies against CGA (Abcam, #92738), GATA2 (Cell Signaling Technology, CST, #4595), Bax (CST, #5023), Bcl2 (CST, #15071), EGFR (CST, #2085), p-EGFR (CST, #3777), ERK (CST, #4695), p-ERK (CST, #4370), AKT (CST, #4691), p-AKT (CST, #4060), GAPDH (CST, #5174), and β-actin (Proteintech, #66009-1) were used. The secondary antibodies used were anti-mouse IgG HRP-linked antibody (CST, #7076) or anti-rabbit IgG HRP-linked antibody (CST, #7074), both of which were used at a 1:1000 dilution.

### Luciferase reporter assay

The miRNA 3′-UTR luciferase reporter vectors were constructed as previously described (38). The binding site of miR-107 targeted to the 3′-UTRs of CGA and GATA2 was predicted using the TargetScan and RNAhybrid public databases. The CGA WT (5′-AGUCUGUGAUGCUGCU-3′), CGA mut-type (5′-CTGTCTTGCGACTGATG-3′), and GATA2 WT (5′-UGGCUUUGCGAGCUGCU-3′) and GATA2 mut-type (5′-TTTGAGGCTACCATGATG-3′) expression vectors were subsequently cloned and inserted into the psiCHECK-2 reporter vector. To conduct the luciferase reporter assay, HEK293T cells were first plated in 24-well plates and then cotransfected with miR-107, the miR-107 inhibitor and its control, and the psiCHECK2-CGA or psiCHECK2-GATA2 vectors with or without the 3′-UTR *via* Lipofectamine 2000 transfection agent. The firefly and Renilla luciferase activities were then measured using the Dual-Luciferase Reporter Assay System (Promega). The firefly luciferase activity was then normalized to the Renilla activity and is presented as the relative luciferase activity to demonstrate the level of activity of the luciferase reporters. The whole 3′-UTR sequence to the constructed vectors of GATA2 was used (https://www.ncbi.nlm.nih.gov/nuccore/NM_001145661.2).

### Cell viability measurement

Cells were successfully seeded in a 96-well plate prior to transfection with miR-107 or miR-ctrl, followed by exposure to chemotherapeutic drugs to induce a proliferative response. A Cell Counting Kit-8 assay (Yeasen) was then utilized to determine the extent of cellular proliferation. This assay involved the incubation of cells with a mixture of cell counting solution and Dulbecco’s modified Eagle’s medium at a ratio of 1:10 at 37 °C for 2 h. After this process, absorbance readings were collected at a wavelength of 450 nm utilizing a Thermo Fisher Scientific Varioskan Flash multimode reader.

A LIVE/DEAD viability/cytotoxicity kit (Thermo Fisher Scientific) was used according to the manufacturer’s instructions. Briefly, the procedure involved seeding cells in 24-well plates, cotransfecting them with miR-107 or miR-ctrl, and then administering chemotherapeutic agents. Following a 48-h incubation period, the cells were subsequently stained in accordance with the manufacturer’s instructions and examined under a fluorescence microscope.

### Apoptosis analysis

The relative proportion of apoptotic cells was evaluated by staining with an Annexin V-FITC Early Apoptosis Detection Kit (CST). After cotransfection with miR-107 or miR-ctrl and treatment with chemotherapeutic drugs, the cells were harvested and resuspended in staining buffer. Subsequently, the cells were analyzed using flow cytometry, and the data were processed and interpreted by EXPO32 ADC software (Beckman Coulter, https://www.beckmancoulter.cn/). Annexin V-FITC–positive and propidium iodide–negative cells were deemed to have undergone apoptosis.

### IC50 assay

To conduct the IC50 assay, 4000 cells were seeded into each well of a 96-well plate and subsequently transfected with either miR-107 or miR-ctrl. The cells were then treated with different concentrations of chemotherapeutic drugs for 48 h, after which they were incubated with a cell counting solution at 37 °C for 2 h. The chemiluminescence intensity measured by a Bio-Rad instrument was used to determine cell proliferation.

### Immunohistochemistry

The immunohistochemical assay was conducted using a diaminobenzidine staining kit (Zhongshan Goldenbridge Biotechnology). Tissue sections were preprocessed by deparaffinization using xylene and 100%-75% ethanol solutions, and antigen retrieval was performed by heating and immersing the sections in an appropriate buffer, followed by cooling at room temperature. Endogenous peroxidase activity was blocked using 3% H_2_O_2_ solution. The sections were incubated with primary antibodies at 4 °C overnight and then with secondary HRP-conjugated goat anti-rabbit or goat anti-mouse antibodies for 1 h. After staining with DAB and hematoxylin, the sections were dehydrated in a gradient of 75%-100% ethanol solutions and xylene. The slides were then mounted with mounting medium and scanned using 3D HISTECH. Additionally, the sections were subjected to H&E staining for histological analysis. Primary antibodies against the following proteins were utilized: Ki67 (CST, #12202, 1:200), PCNA (CST, #13110, 1:1000), cleaved caspase-3 (CST, #9661, 1:400), Bax (Immunoway, #YT0455, 1:200), CGA (Proteintech, #25014, 1:500), GATA2 (Proteintech, #11103, 1:100), p-EGFR (CST, #3777, 1:200), p-ERK (CST, #4370, 1:200), and p-AKT (Immunoway, #YP0006, 1:200). The IHC score was calculated based on both the intensity and extent of the target molecule's staining in each specimen. The intensity of staining was scored as follows: 0, no staining detected; 1, weakly stained cells; 2, moderately stained cells; and 3, strongly stained cells. The extent of staining was assessed based on the percentage of positively stained tumor cells in each slide and was scored as follows: 0, no positive cells observed; 1, fewer than 10% positive cells; 2, 10 to 50% positive cells; and 3, more than 50% positive cells.

### Multiplex immunofluorescence staining

Multiplex immunofluorescence staining was performed using an Opal 7-color IHC kit (Akoya Biosciences) as previously described (39). The sections were incubated at a constant temperature of 60 to 65 °C for 2 h. After dewaxing and hydration with xylene and gradient ethanol solutions, the sections were fixed with 10% formalin for 10 to 20 min. The antigens were retrieved in a microwave oven with EDTA (pH = 9.0) antigen repair solution and subsequently treated with 3% H_2_O_2_ for 10 to 15 min to block endogenous peroxidase. After blocking for 10 min, the primary antibody was added, and the cells were incubated for 1 h. Next, the secondary antibody was added and incubated for 10 min. Opal staining was then performed utilizing the fluorescein tyramine signal amplification technique. The antigen repair treatment was repeated in a microwave oven to remove the primary and secondary antibodies. Then, blocking buffer, primary antibodies, and secondary antibodies were added, and the sections were treated with tyramine signal amplification. This process was repeated until all of the antigens had been labeled with a different fluorescence signal. After elution, the sections were subjected to 4′6-diamidino-2-phenylindole staining for 5 min and then sealed and fixed using anti-quench fluorescence. Finally, multispectral analysis was carried out through calculating IHC score which counts positive cells per mm^2^ of regional tissue area using the PerkinElmer Vectra 3 Imaging system. The following target proteins were labeled with antibodies: CGA (Abcam, #92738, 1:100); GATA2 (CST, #4595, 1:200); EGFR (CST, #2085, 1:200); p-EGFR (CST, #3777, 1:400); and CK (CST, #17171, 1:100).

### Production of the miR-107 prodrug

The successful expression and purification of a recombinant Sephadex aptamer-tagged methionyl-tRNA/miR-107 (the miR-107 prodrug) and control prodrug, a Sephadex aptamer-tagged methionyl-tRNA scaffold (tRNA/MSA), were conducted as previously described (26, 27, 28). Briefly, miR-107 and MSA were transformed into *Escherichia coli* cells. To separate the target RNAs from the total bacterial RNA, the NGC QUEST 10PLUS FPLC system (Bio-Rad) was used. This system was initially equilibrated with buffer A (10 mM sodium phosphate, pH 7.0) at a constant flow rate of 2.5 ml/min for 4.4 min, followed by gradient elution with 64% buffer B (buffer A + 1 M sodium chloride, pH 7.0) for 10 min, 64 to 78% buffer B for 8 min, and then 100% buffer B for 3 min. The FPLC traces were monitored at 260/280 nm using a UV/Vis detector. Following confirmation of the target RNA by urea-PAGE analyses, the fractions were pooled, precipitated with ethanol, desalted, and concentrated/dissolved in nuclease-free water using an Amicon Ultra2 ml centrifugal filter (30 kDa; EMD Millipore). The RNA purity was verified by high-performance liquid chromatography. Recombinant RNAs with purities greater than 97% were used in the present study. Purified MSA/miR-107 and tRNA/MSA were intratumorally injected into mice using in vivo-jetRNA+ (Polyplus Transfection) reagent. Complexes were formed with purified MSA/miR-107 and *in vivo* jetRNA+ in complexation buffer for intratumoral injection. The prepared complexes (20 μl per mouse) were slowly injected into the subcutaneous tumor within 15 min.

### *In vivo* drug resistance assay

To conduct *in vivo* drug resistance assays, male BALB/c nude mice, averaging approximately 20 g in weight, were obtained from Beijing Vital River Laboratory Animal Technology. SGC7901^ADR^ cells were cultivated and then implanted subcutaneously into the posterior flanks of the mice (5 × 10^6^ tumor cells/150 μl of PBS per spot; five mice in each group). Once the tumor size reached a predetermined amount (approximately 100 mm^3^), the mice were randomly divided into control and treatment groups. Tumor growth was measured with a bilateral caliper, which is an indicator of drug efficacy. Tumor volume was calculated using the following formula: tumor maximum diameter (*L*) × the right-angle diameter to that axis (*W*)^2^/2. Following a 2-week period of treatment, the mice were sacrificed by CO_2_ inhalation in compliance with institutional ethical guidelines. The size and weight of the tumors, along with measurements of the livers and kidneys, were considered after *postmortem* examination. Tumor growth was assessed through paraffin embedding of the organs. The Air Force Medical University Animal Care Committee approved the study protocol.

### Bioinformatics prediction and analysis

Bioinformatics algorithms, including DIANAmT (http://www.microrna.gr/mited), miRanda (http://www.microrna.org/microrna/getMirnaForm.do), miRDB (http://www.mirdb.org/), miRWalk (http://mirwalk.umm.uni-heidelberg.de/), RNAhybrid (http://bibiserv.techfak.uni-bielefeld.de/rnahybrid/), PICTAR (http://pictar.mdc-berlin.de/), PITA (http://genie.weizmann.ac.il/pubs/mir07/mir07_data.html), and RNA22 (http://cbcsrv.watson.ibm.com/rna22.html), were utilized to screen miRNAs that target both CGA and GATA2. To investigate the association between the prognosis of GC patients and the expression of candidate miRNAs, the Kaplan‒Meier plotter database (https://kmplot.com/analysis/) was utilized.

### Statistical analysis

Statistical analyses were carried out utilizing GraphPad Prism 8, a software package for data analysis and visualization. All quantitative data are presented as the mean ± SD or mean ± SEM. To determine the significance of any differences between the two groups, Student’s *t* test was used. To evaluate the significance of differences among multiple groups, one-way ANOVA and repeated-measures ANOVA with Dunnett’s multiple-comparison test were used. A *p* value less than 0.05 was considered to indicate statistical significance.

## Data availability

The data that support the findings of this study are available in the supplementary file. Further data used and/or analyzed in the study can be directed to the corresponding author on reasonable request.

## Supporting information

This article contains supporting information (40).

## Conflict of interest

The authors declare that they have no conflicts of interest with the contents of this article.

## References

[1] Sung H., Ferlay J., Siegel R.L., Laversanne M., Soerjomataram I., Jemal A. (2021). Global cancer statistics 2020: GLOBOCAN estimates of incidence and mortality worldwide for 36 cancers in 185 countries. CA Cancer J. Clin..

[2] Yuan M., Yang Y., Lv W., Song Z., Zhong H. (2014). Paclitaxel combined with capecitabine as first-line chemotherapy for advanced or recurrent gastric cancer. Oncol. Lett..

[3] Cunningham D., Allum W.H., Stenning S.P., Thompson J.N., Van de Velde C.J., Nicolson M. (2006). Perioperative chemotherapy versus surgery alone for resectable gastroesophageal cancer. N. Engl. J. Med..

[4] Huang H., Yang X.J., Gao R. (2016). Research advances in the mechanisms of gastric cancer multidrug resistance. Zhongguo Yi Xue Ke Xue Yuan Xue Bao.

[5] Chen Z.D., Zhang P.F., Xi H.Q., Wei B., Chen L., Tang Y. (2021). Recent advances in the diagnosis, staging, treatment, and prognosis of advanced gastric cancer: a literature review. Front. Med. (Lausanne).

[6] Oda K., Matsuoka Y., Funahashi A., Kitano H. (2005). A comprehensive pathway map of epidermal growth factor receptor signaling. Mol. Syst. Biol..

[7] Wee P., Wang Z. (2017). Epidermal growth factor receptor cell proliferation signaling pathways. Cancers (Basel).

[8] Arlt A., Muerkoster S.S., Schafer H. (2013). Targeting apoptosis pathways in pancreatic cancer. Cancer Lett..

[9] Wu M., Zhang P. (2020). EGFR-mediated autophagy in tumourigenesis and therapeutic resistance. Cancer Lett..

[10] Schmidt M., Lichtner R.B. (2002). EGF receptor targeting in therapy-resistant human tumors. Drug Resist. Updat..

[11] Rajaram P., Chandra P., Ticku S., Pallavi B.K., Rudresh K.B., Mansabdar P. (2017). Epidermal growth factor receptor: role in human cancer. Indian J. Dent. Res..

[12] Cao T., Lu Y., Wang Q., Qin H., Li H., Guo H. (2022). A CGA/EGFR/GATA2 positive feedback circuit confers chemoresistance in gastric cancer. J. Clin. Invest..

[13] Vokes E.E., Chu E. (2006). Anti-EGFR therapies: clinical experience in colorectal, lung, and head and neck cancers. Oncology (Williston Park).

[14] Slack F.J., Chinnaiyan A.M. (2019). The role of non-coding RNAs in oncology. Cell.

[15] Lam J.K., Chow M.Y., Zhang Y., Leung S.W. (2015). siRNA versus miRNA as therapeutics for gene silencing. Mol. Ther. Nucleic Acids.

[16] Bartel D.P. (2004). MicroRNAs: genomics, biogenesis, mechanism, and function. Cell.

[17] Daka A., Peer D. (2012). RNAi-based nanomedicines for targeted personalized therapy. Adv. Drug Deliv. Rev..

[18] Fu Q., Cheng J., Zhang J., Zhang Y., Chen X., Xie J. (2017). MiR-145 inhibits drug resistance to Oxaliplatin in colorectal cancer cells through regulating G protein coupled receptor 98. Zhonghua Wei Chang Wai Ke Za Zhi.

[19] Wang Q., Cao T., Guo K., Zhou Y., Liu H., Pan Y. (2020). Regulation of integrin subunit alpha 2 by miR-135b-5p modulates chemoresistance in gastric cancer. Front. Oncol..

[20] Chen Z., Zhang L., Xia L., Jin Y., Wu Q., Guo H. (2014). Genomic analysis of drug resistant gastric cancer cell lines by combining mRNA and microRNA expression profiling. Cancer Lett..

[21] Chen H.A., Li C.C., Lin Y.J., Wang T.F., Chen M.C., Su Y.H. (2021). Hsa-miR-107 regulates chemosensitivity and inhibits tumor growth in hepatocellular carcinoma cells. Aging (Albany NY).

[22] Gao S.J., Ren S.N., Liu Y.T., Yan H.W., Chen X.B. (2021). Targeting EGFR sensitizes 5-Fu-resistant colon cancer cells through modification of the lncRNA-FGD5-AS1-miR-330-3p-Hexokinase 2 axis. Mol. Ther. Oncolytics.

[23] Zhou Z., Lin Z., He Y., Pang X., Wang Y., Ponnusamy M. (2018). The long noncoding RNA D63785 regulates chemotherapy sensitivity in human gastric cancer by targeting miR-422a. Mol. Ther. Nucleic Acids.

[24] Shang Y., Feng B., Zhou L., Ren G., Zhang Z., Fan X. (2016). The miR27b-CCNG1-P53-miR-508-5p axis regulates multidrug resistance of gastric cancer. Oncotarget.

[25] Wang Q., Shi S., He W., Padilla M.T., Zhang L., Wang X. (2014). Retaining MKP1 expression and attenuating JNK-mediated apoptosis by RIP1 for cisplatin resistance through miR-940 inhibition. Oncotarget.

[26] Tu M.J., Ho P.Y., Zhang Q.Y., Jian C., Qiu J.X., Kim E.J. (2019). Bioengineered miRNA-1291 prodrug therapy in pancreatic cancer cells and patient-derived xenograft mouse models. Cancer Lett..

[27] Chen Q.X., Wang W.P., Zeng S., Urayama S., Yu A.M. (2015). A general approach to high-yield biosynthesis of chimeric RNAs bearing various types of functional small RNAs for broad applications. Nucleic Acids Res..

[28] Ho P.Y., Duan Z., Batra N., Jilek J.L., Tu M.J., Qiu J.X. (2018). Bioengineered noncoding RNAs selectively change cellular miRNome profiles for cancer therapy. J. Pharmacol. Exp. Ther..

[29] Luo Y., Hua T., You X., Lou J., Yang X., Tang N. (2019). Effects of MiR-107 on the chemo-drug sensitivity of breast cancer cells. Open Med. (Wars).

[30] Liang Y., Zhu D., Hou L., Wang Y., Huang X., Zhou C. (2020). MiR-107 confers chemoresistance to colorectal cancer by targeting calcium-binding protein 39. Br. J. Cancer.

[31] Su P.F., Song S.Q. (2020). Regulation of mTOR by miR-107 to facilitate glioma cell apoptosis and to enhance cisplatin sensitivity. Eur. Rev. Med. Pharmacol. Sci..

[32] Khvorova A., Watts J.K. (2017). The chemical evolution of oligonucleotide therapies of clinical utility. Nat. Biotechnol..

[33] Robbins M., Judge A., MacLachlan I. (2009). siRNA and innate immunity. Oligonucleotides.

[34] Pereira P., Pedro A.Q., Queiroz J.A., Figueiras A.R., Sousa F. (2017). New insights for therapeutic recombinant human miRNAs heterologous production: rhodovolum sulfidophilum vs Escherichia coli. Bioengineered.

[35] Du J., Shi Y., Pan Y., Jin X., Liu C., Liu N. (2005). Regulation of multidrug resistance by ribosomal protein l6 in gastric cancer cells. Cancer Biol. Ther..

[36] Li K., Sun Z., Zheng J., Lu Y., Bian Y., Ye M. (2013). In-depth research of multidrug resistance related cell surface glycoproteome in gastric cancer. J. Proteomics.

[37] Scheper R.J., Broxterman H.J., Scheffer G.L., Kaaijk P., Dalton W.S., van Heijningen T.H. (1993). Overexpression of a M(r) 110,000 vesicular protein in non-P-glycoprotein-mediated multidrug resistance. Cancer Res..

[38] Zhao X., He L., Li T., Lu Y., Miao Y., Liang S. (2014). SRF expedites metastasis and modulates the epithelial to mesenchymal transition by regulating miR-199a-5p expression in human gastric cancer. Cell Death Differ..

[39] He Y., Han Y., Fan A.H., Li D., Wang B., Ji K. (2022). Multi-perspective comparison of the immune microenvironment of primary colorectal cancer and liver metastases. J. Transl. Med..

[40] Liu S., Wang Z., Zhu R., Wang F., Cheng Y., Liu Y. Three differential expression analysis methods for rna sequencing: limma, EdgeR, DESeq2. *J. Vis. Exp*. 10.3791/62528.

