# Supplementary figures and images for: miR-107 reverses the multidrug resistance of gastric cancer by targeting the CGA/EGFR/GATA2 positive feedback circuit
Source: J Biol Chem. 2024 Jul 2;300(8):107522. doi: 10.1016/j.jbc.2024.107522 (PMC11345541; doi:10.1016/j.jbc.2024.107522)

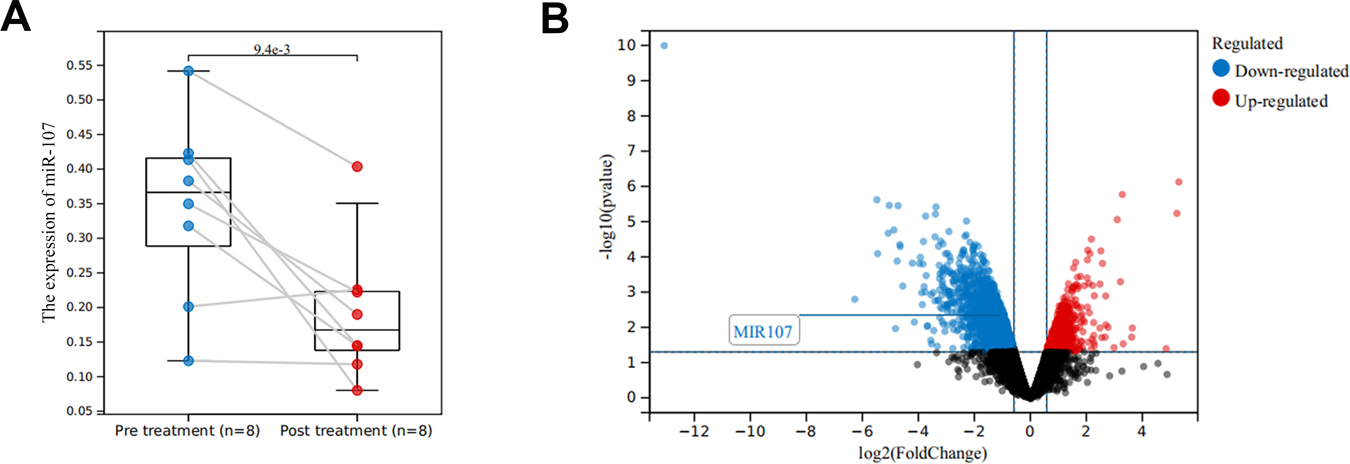

Supplement: Supplementary Figure 1 [file figs1.jpg]

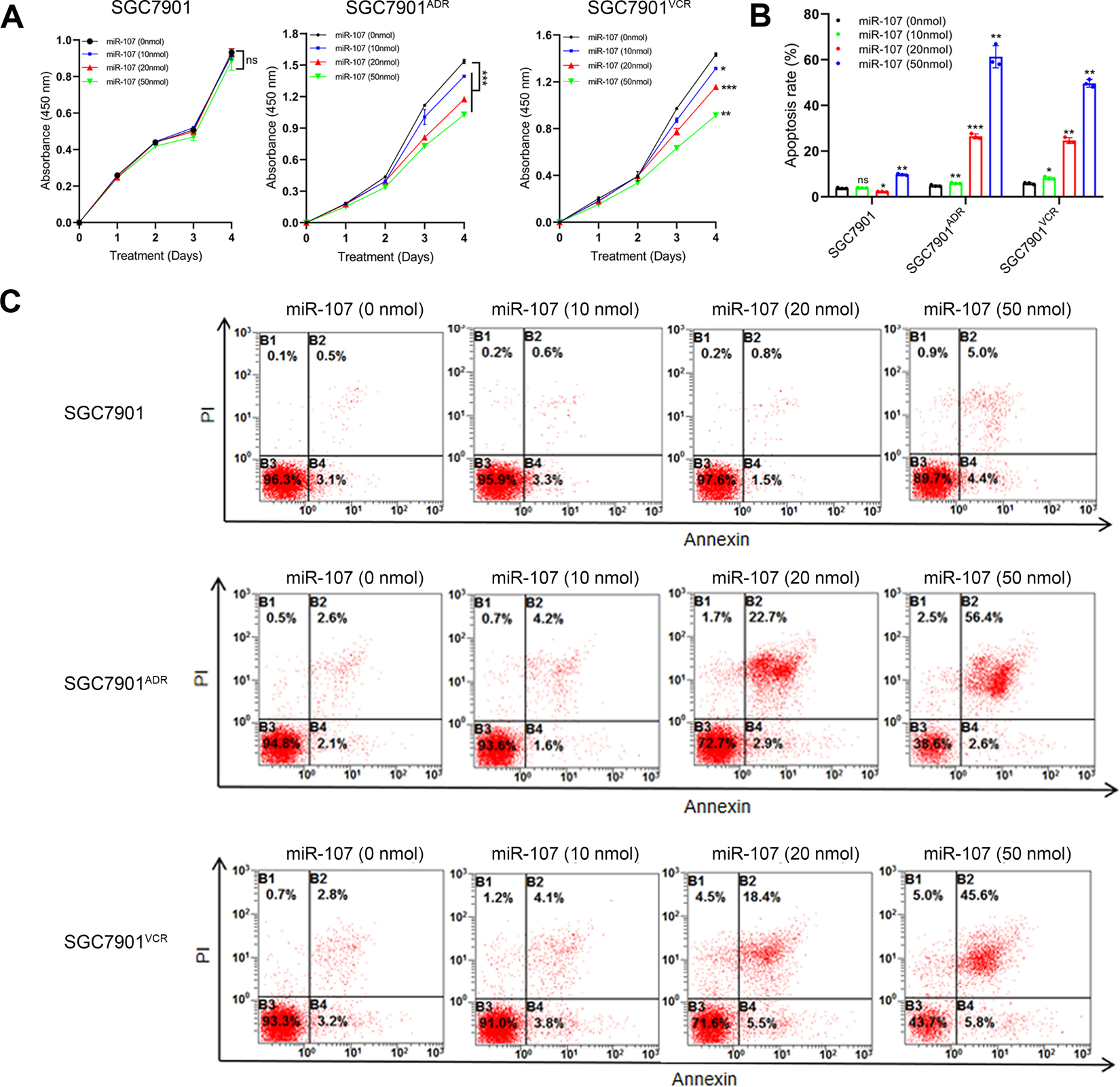

Supplement: Supplementary Figure 2 [file figs2.jpg]
