# Supplementary material for: miR-107 reverses the multidrug resistance of gastric cancer by targeting the CGA/EGFR/GATA2 positive feedback circuit
Source: J Biol Chem. 2024 Jul 2;300(8):107522. doi: 10.1016/j.jbc.2024.107522 (PMC11345541; doi:10.1016/j.jbc.2024.107522)
Supplement: Supporting figure legends [file mmc4.docx]

**Supplementary Figure 1. The expression of miR-107 in GEO datasets (GSE30070 and GSE49052).**

(A) miR-107 expression in the GSE30070 dataset, which consists of paired data before andafter CF(cisplatin/fluorouracil) chemotherapy. A paired t test was used to analyze the statistical significancebetween two groups. (B) miR-107 expression in paired GSE49052 gastric cancer cells before andafter drugtreatment. The limma method was used to analyze the statistical significance between two groups (40).

**Supplementary Figure 2. The effect of miR-107 alone on MDR-GC cells *in vitro*.**

(A) Proliferation of SGC7901, SGC7901^ADR^ and SGC7901^VCR^ cells after transfection with different concentrations of miR-107. (B-C) Apoptosis of SGC7901, SGC7901^ADR^ and SGC7901^VCR^ cells after transfection with different concentrations of miR-107. The data in the bar plots are expressed as the mean ± S.D. (n=3). Significant differences were assessed by repeated-measures ANOVA (A) and one-way ANOVA (B). *P< 0.05, **P< 0.01, ***P< 0.001. ns, not significant.
